# Creative Mindsets: Scale Validation in the Chinese Setting and Generalization to the Real Workplace

**DOI:** 10.3389/fpsyg.2020.00463

**Published:** 2020-03-26

**Authors:** Yiyong Zhou, Wa Yang, Xinwen Bai

**Affiliations:** ^1^CAS Key Laboratory of Behavioral Science, Institute of Psychology, Chinese Academy of Sciences, Beijing, China; ^2^Department of Psychology, University of Chinese Academy of Sciences, Beijing, China; ^3^School of Labor and Human Resources, Renmin University of China, Beijing, China

**Keywords:** growth/fixed creative mindsets, scale development, Chinese settings, implicit theories, creativity

## Abstract

Creative mindsets reflect the implicit beliefs individuals hold regarding the nature of creativity as innate (i.e., fixed mindset) or malleable (i.e., growth mindset). Karwowski (2014) developed the Creative Mindsets Scale (CMS), in which fixed and growth creative mindsets were each measured with five items. Across three studies, the current study aimed to examine its psychometric properties in Chinese settings and to explore to what extent effects of creative mindsets on creativity were generalized to the real workplace. Based on the survey data of 216 college students (Study 1) and 205 full-time employees (Study 2) in China, results consistently indicated that a two-factor structure, in which both types of creative mindsets were independent of each other, was confirmed. Measures of both types of creative mindsets were of satisfactory psychometric features in terms of reliability (internal consistency) and validity (construct, convergent, and discriminant validities). Furthermore, Study 1 provided evidence for the incremental validity of creative mindsets beyond mindsets of intelligence in explaining creative personal identity and creative self-efficacy. Based on a third independent sample consisting of 282 full-time employees from several Chinese companies, Study 3 further demonstrated that measures of creative mindsets could predict employees’ creative performance as rated by their supervisors, lending additional support for their generalizability to the real workplace. Moreover, growth mindset, but not fixed mindset, was significantly related to creative performance, and such an effect was mediated by effort. The present study contributes to the creative mindset literature by cross-validating the CMS’s psychometric properties in a new setting and empirically establishing the link between creative mindsets and employees’ creativity in the real workplace.

## Introduction

It’s long been documented that people hold different beliefs about the malleability of human general attributes such as intelligence (Dweck, 1999; Blackwell et al., 2007), personality (Chiu et al., 1997b), morality (Chiu et al., 1997a), and even many specific attributes like willpower (Job et al., 2010), interest (O’Keefe et al., 2018), or emotion (Tamir et al., 2007). These beliefs are the manifestation of their implicit theories or mindsets. Dweck and Leggett (1988) and Dweck (1999, 2006) have identified two distinct mindsets. Those with an entity theory (or fixed mindset) believe that human basic traits are fixed and cannot be improved or changed much. In contrast, those with an incremental theory (or growth mindset) believe that such attributes are malleable and can be developed to a great extent. Existing literature consistently demonstrates that a growth mindset leads to a higher level of goal achievement (Blackwell et al., 2007) than a fixed mindset, especially when facing adverse situations (Aronson et al., 2002; Claro et al., 2016). Those holding the growth mindset have higher subjective well-being and less depressive symptoms (Tamir et al., 2007; Zeng et al., 2016; Bernecker et al., 2017). The advantage of a growth mindset over a fixed mindset is mainly attributed to the belief that one can always overcome setbacks and achieve goals by hard work (Dweck, 2006). As a result, individuals holding the growth mindset tend to adopt learning goals rather than performance goals (Burnette et al., 2013), exert more efforts to achieve goals (Schumann et al., 2014), are more resilient and persistent while facing setbacks (Aronson et al., 2002), and recover better from failures (Howe and Dweck, 2016).

People also hold an implicit theory of creativity, or creative mindsets, regarding its fixed or malleable feature. Defined as “beliefs about the stable-versus-malleable character and the nature of creativity” (Karwowski, 2014, p. 62), the creative mindsets reflect the perceptions people hold implicitly regarding creativity as something that is innate (fixed mindset) or can be developed (growth mindset). Preliminary evidence indicates that a growth creative mindset is associated with higher creative self-efficacy (CSE), creative identity (Hass et al., 2016), and better performance in creative problem-solving tasks (Royston and Reiter-Palmon, 2017) and insight problems (Karwowski, 2014); a fixed creative mindset is, on the other hand, negatively related to these outcome variables.

Still in its infancy, a central theme of creative mindsets research is whether fixed and growth mindsets form two opposites of the same continuum or are two distinct, independent constructs. Following Dweck and colleagues, some researchers treat these two creative mindsets as mutually exclusive alternatives (e.g., Makel, 2009; O’Connor et al., 2013). According to this view, people holding a growth mindset are exactly those who do not endorse a fixed mindset, and vice versa. Other researchers (e.g., Karwowski, 2014; Hass et al., 2016; Karwowski et al., 2019b), however, argue that fixed and growth creative mindsets are not necessarily opposite but, rather, independent dimensions. In another word, it is possible for individuals to endorse both fixed and growth creative mindsets simultaneously.

To fully capture the dual-dimension feature of creative mindsets, Karwowski (2014) developed the Creative Mindsets Scale (CMS), in which each creative mindset is measured with five items. The scale has been demonstrated to adequately meet psychometric criteria (Karwowski, 2014). Moreover, accumulative evidence has recently been more in favor of the independent-dimension view than the opposite-end view (Puente-Díaz and Cavazos-Arroyo, 2017b; Karwowski et al., 2019b). Items of this newly developed scale, in terms of factor loadings, outperformed those directly borrowed from items of Dweck’s (1999) implicit theories of intelligence scale, with the word “creativity” replacing “intelligence” (Hass et al., 2016).

While this scale was originally constructed in Poland, it has subsequently been examined in the United States (Hass et al., 2016; Royston and Reiter-Palmon, 2017), Germany (Tang et al., 2016), Thailand (Intasao and Hao, 2018), and Korea (Lee and Choi, 2014). Results of these cross-cultural validations indicate that the scale has satisfactory psychometric properties and can be generalized to other contexts. To our best knowledge, there is no published paper addressing its validation and application in the Chinese context. Given the important role culture plays in shaping people’s implicit theories of creativity (Niu and Sternberg, 2002; Loewenstein and Mueller, 2016), it’s of great importance to examine whether creativity is also perceived as fixed or malleable by the Chinese people and to explore how creative mindsets are inter-related with other constructs in the Chinese setting. Furthermore, although researchers have recently begun to examine the effects of creativity mindsets on individuals’ creative performance, most of the studies only targeted student samples (e.g., Royston and Reiter-Palmon, 2017; Intasao and Hao, 2018) or relied on participants’ self-report of their performance on given creativity tasks (e.g., O’Connor et al., 2013). The question of how creative mindsets will influence employees’ daily creativity in the workplace remains unanswered.

Therefore, the main purpose of current study was to validate the CMS’s psychometric properties in the Chinese setting and to examine its generalizability to the workplace. Specifically, the first two studies were conducted to test its psychometric properties in terms of factor structure, reliability, and convergent and discriminant validity by using a sample of college students (Study 1) and an independent sample of full-time employees (Study 2), respectively. Further, Study 3 aimed to explore whether creative mindsets were associated with individuals’ creativity in the real workplace and to reveal the mediating mechanism as well. By adopting a multi-sample strategy, we had the chance to cross-validate its psychometric properties and to extend its application in diverse settings. All of the procedures performed in studies involving human participants were approved by the Ethics Committee of the Institute of Psychology, Chinese Academy of Sciences. Studies 2 and 3 were also approved by the corresponding human resource department of the company at which the survey was conducted.

## Study 1

The aim of Study 1 was twofold: (a) to examine the construct validity of the CMS with a specific focus on the relationship between two types of creative mindsets, namely, whether fixed and growth mindsets were independent dimensions or two ends of the same continuum; and (b) to test its discriminant and convergent validity by assessing the relationships between creative mindsets and other relevant constructs. Aligned with assertions that mindsets shape self-beliefs and that self-beliefs form a basis for mindset development (Karwowski and Brzeski, 2017), we postulated that creative mindsets were correlated yet distinct from self-constructs such as creative identity (Jaussi et al., 2007), CSE (Tierney and Farmer, 2002), and mindsets of intelligence (Dweck and Leggett, 1988; Dweck, 1999, 2006). We relied mainly on confirmative factor analysis (CFA) to examine the CMS’s psychometric properties. Furthermore, we examined discriminant validity between mindsets of creativity and intelligence by testing the incremental predictive effect of creative mindsets on creativity-specific constructs (e.g., creative identity and CSE).

### Mindsets of Creativity and Intelligence

As illustrated in the literature, implicit theories, or mindsets, are domain specific (Dweck et al., 1995). People may hold different theories toward different human attributes. For example, the same person who believes that intelligence is fixed can see morality or personality as malleable. Likewise, implicit theories of intelligence and creativity are distinct and independent from each other (Sternberg, 1985; O’Connor et al., 2013; Plucker et al., 2017).

Sternberg (1985) pioneered in the attempt to extract people’s implicit theories of creativity from those about intelligence. By focusing on people’s personal definition of each construct, Sternberg (1985) found that people held different and clear implicit theories about creativity and intelligence conceptually. To be more specific, people’s implicit theories for each construct were systematic and distinct from each other (indication of discriminant validity), and people could use these implicit theories to evaluate levels of intelligence or creativity of others or of themselves accurately (evidence of convergent validity). However, a study by Plucker et al. (2017) showed that for Asians, there was a considerable overlap between implicit theories of intelligence and creativity. It appeared that Asian participants had difficulties in distinguishing intelligence from creativity because problem-solving ability was critical to each set of their implicit theories. Inconsistent findings suggest that further research is necessary to explore the relationship between mindsets of intelligence and creativity.

O’Connor et al. (2013) adopted another approach to statistically separate the validity of creative mindsets from that of mindsets of intelligence in predicting creativity-relevant outcomes. Its underlying logic was that mindsets were domain specific and that only beliefs in creativity could predict creativity criteria. As expected, their findings proved that a fixed creative mindset was consistently and significantly associated with a lower level of creativity across a series of criteria, such as interest/engagement in creative activities, self-reported creativity, and performance of an unusual uses task. On the contrary, a fixed mindset of intelligence was not significantly related to any of these creativity outcomes. However, fixed and growth mindsets were treated as opposite to each other in this study. Therefore, it’s not clear whether each type of creative mindset, when defined as an independent construct, has incremental validity or is distinct from its counter mindset of intelligence.

### Creative Mindsets and CSE

Defined as “the belief one has the ability to produce creative outcomes” (Tierney and Farmer, 2002, p. 1138), CSE is consistently and positively associated with a higher level of creativity in different types of measures (for evidence supporting the relationship between CSE and creativity, see meta-analyses by Liu et al., 2016; Haase et al., 2018). CSE facilitates creativity mainly because individuals with high CSE tend to adopt a mastery goal, be more willing to initiate creative actions, and be more persistent in creative endeavors (Tierney and Farmer, 2002; Beghetto, 2006; Gong et al., 2009; Tierney and Farmer, 2011; Pretz and Nelson, 2017).

Researchers have recently begun to explore the relationship between creative mindsets and CSE. Preliminary studies indicate that a growth creative mindset is positively correlated with CSE, while the correlation between a fixed creative mindset and CSE is either non-significant or negative (Karwowski, 2014; Puente-Díaz and Cavazos-Arroyo, 2017a; Royston and Reiter-Palmon, 2017). Some researchers go further to test a causal relationship between creative mindsets and CSE (Karwowski, 2014; Royston and Reiter-Palmon, 2017). Although such an argument is restricted by the cross-sectional design, when taken together, it provides evidence that CSE could serve as a criterion to test the construct validity of creative mindsets. Despite them both being key determinants of creative outcomes, they target different aspects and are conceptually different. CSE is the belief in one’s ability to be creative (Tierney and Farmer, 2002; Beghetto, 2006); creative mindsets reflect one’s perception about the nature of creativity (O’Connor et al., 2013; Karwowski, 2014). Hass et al. (2016) empirically demonstrated that measures of creative mindsets could be disentangled from that of CSE. Thus, both conceptual and empirical evidence suggests that creative mindsets and CSE should be related yet discriminated from each other. In order to test the construct validity of creative mindsets in the Chinese setting, we included CSE in our study.

### Creative Mindsets and Creative Personal Identity

In a similar vein, creative mindsets are conceptually distinct from creative personal identity (CPI), which is defined as an individual’s belief about the importance of creativity to his/her self-description (Jaussi et al., 2007). An array of studies have exhibited a positive link between CPI and creative performance (Farmer et al., 2003; Jaussi et al., 2007; Tierney and Farmer, 2011). Conceptually, CPI is closely related to creative mindsets (Pretz and Nelson, 2017), but research on their correlations is relatively limited and shows a tendency similar to those of CSE. For example, using samples of college students from different cultural backgrounds, one recent study found that CPI was positively related to a growth creative mindset while negatively related to a fixed mindset (Pretz and Nelson, 2017). Meanwhile, as indicated in a study by Hass et al. (2016), creative mindsets and CPI emerged as independent factors in both exploratory and CFAs. Accordingly, we included CPI as another criterion to examine the construct validity of creative mindsets.

### Participants

A total of 216 participants were recruited through online advertisement in several universities in China (mainly from Beijing). They were invited to complete an online self-report questionnaire. Informed consent was obtained by a yes/no screen question prior to the survey, and all participants were informed that the survey was anonymous and that they could withdraw anytime during the process. The survey took an average of 10 min, and we included two instructed response items to avoid careless responses (Meade and Craig, 2012). All participants passed this test, indicating sufficient efforts when answering the online survey. After completing the survey, they were briefed and thanked, with 10 RMB as compensation for their participation.

Among all participants, 137 were female (63.4%), and 79 were male (36.6%). Age ranged from 17 to 30, with a mean of 22.99 years old (*SD* = 2.31). A majority of them were master students (*n* = 120; 55.6%), 77 of them were undergraduate students (35.6%), and the rest were doctoral students (*n* = 19; 8.8%). Nearly half of them were majors in science, technology, engineering, and math (STEM) (*n* = 103; 47.7%), and the other half were in the humanities and social sciences (*n* = 113; 52.3%).

### Measures

All questionnaires were the original English version. To ensure accuracy, two bilingual experts translated all English items into Chinese following translation and back-translation procedures (Brislin, 1986).

#### Creative Mindsets

We used Karwowski’s (2014) CMS to measure participants’ perceptions of the nature of creativity. This scale consists of two subscales, each with five items. Participants were instructed to indicate the extent to which these items reflected their belief in the fixed (e.g., “You either are creative or you are not—even trying very hard you cannot change much.”) or malleable nature of creativity (e.g., “Everyone can create something great at some point if he/she is given appropriate conditions.”), on a five-point Likert scale ranging from 1 (definitely not) to 5 (definitely yes). For this study, Cronbach’s α was 0.84 for the fixed creative mindset and 0.70 for the growth creative mindset.

#### Mindsets About Intelligence

We measured participants’ mindsets of intelligence using Dweck’s (1999) eight-item Intelligence Mindset Scale. Four items test the extent to which one believes that intelligence is fixed and cannot be changed (e.g., “Intelligence is something that cannot be changed very much.”), and the other four capture people’s belief about intelligence that is changeable and can be improved (e.g., “Everyone can improve their intelligence to a certain level.”). Participants responded on a six-point Likert scale ranging from 1 (strongly disagree) to 6 (strongly agree). For this study, Cronbach’s α was 0.89 for the fixed mindset of intelligence and 0.92 for the growth mindset.

#### Creative Personal Identity

Creative identity was tested by Burgmer et al.’s (2019) four-item scale. An example item is “To be a creative person is an important part of my identity.” Participants responded on a seven-point Likert scale from 1 (strongly disagree) to 7 (strongly agree), α = 0.77.

#### Creative Self-Efficacy

We used a CSE scale (Tierney and Farmer, 2002) to examine participants’ perception about their creative ability (e.g., “I have confidence in my ability to solve problems creatively.”). This scale has been well-validated in the Chinese setting (Gong et al., 2009). Participants responded on a seven-point Likert scale (1 = strongly disagree, 7 = strongly agree; α = 0.87).

### Results and Discussion

We relied on CFA to examine the construct validity of the CMS. Specifically, we compared the fitness of the hypothesized model with the alternative one in terms of Comparative Fit Index (CFI), Tucker Lewis Index (TLI), Root Mean Square Error of Approximation (RMSEA), and Standardized Root Mean Square Residual (SRMR). Based on CFA results, factor structure was first examined, and convergent and discriminant validity were further assessed according to composite reliability (CR) and average variance extracted (AVE) (Fornell and Larcker, 1981; Raykov, 1997). Moreover, a series of hierarchical multiple regressions were conducted to explore the incremental validity of creative mindsets over mindsets of intelligence (Hunsley and Meyer, 2003).

#### Confirmative Factor Analysis

The CMS was originally developed as a two-factor structure, of which fixed and growth creative mindsets were two independent dimensions (Karwowski, 2014; see also, Karwowski et al., 2019b). Accordingly, we constructed a two-factor model and tested it by using CFA with the maximum likelihood estimation in Mplus 7.3. The model yielded a satisfactory fit with the data (χ^2^_(34)_ = 58.34, *p* < 0.01, CFI = 0.96, TLI = 0.95, RMSEA = 0.058, 90% CI = [0.031, 0.082], SRMR = 0.054).[[Au Query:]] On the contrary, there was an alternative single-factor model in which all items loading on one factor did not fit with the data at all (χ^2^_(35)_ = 173.52, *p* < 0.001, CFI = 0.79, TLI = 0.73, RMSEA = 0.135, 90% CI = [0.116, 0.156], SRMR = 0.103). The advantage of the two-factor solution over the one-factor model was further supported by a significant chi-square difference (Δχ^2^_(1)_ = 115.18, *p* < 0.001) between them.

**FIGURE 1:**
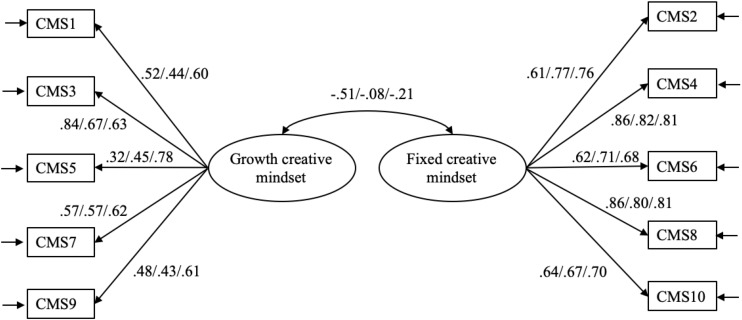
Results of confirmative factor analysis (CFAs) for the two-factor models of the Creative Mindsets Scale (CMS) (Studies 1–3). Standardized factor loadings. All factor loadings are significant at the 0.001 level. The first, second, and third figures correspond to item factor loading or latent correlation in Study 1, Study 2, and Study 3, respectively.

All factor loadings and the correlation between the two latent variables of the two-factor model are depicted in Figure 1. As shown in Figure 1, factor loadings were all significant at the 0.001 level and were above 0.50 except for two items (item 5 and item 9) measuring growth mindset. No modification index was suggested for any cross-loading, indicating that each item was the exclusive indicator of its intended latent construct. Latent correlation was negative and moderate (*r* = −0.51, *t* = −7.91, *p* < 0.001), indicating that fixed and growth creative mindsets were correlated but different constructs. Taking these together, the hypothesized two-factor structure was supported.

#### Convergent and Discriminant Validity

We assessed the convergent and discriminant validity of the CMS via CR, AVE, and shared variances (Hair et al., 2009). We conducted a CFA with all six constructs in the same model. The hypothesized six-factor model had an adequate fit with the data (χ^2^_(284)_ = 549.26, *p* < 0.001, CFI = 0.91, TLI = 0.90, RMSEA = 0.066, 90% CI = [0.057, 0.074], SRMR = 0.065). We subsequently calculated the aforementioned statistics based on CFA results. According to Hair et al. (2009), convergent validity is adequate when CR is 0.70 or higher and is more satisfactory if coupled with an AVE of 0.50 or greater. As shown in Table 1, both fixed (CR = 0.85) and growth creative mindsets (CR = 0.70) reached the 0.70 cutoff of CR, suggesting an acceptable convergent validity for both measures. In addition, fixed creative mindset (AVE = 0.53) exceeded the 0.50 cutoff of AVE, indicating its stronger convergent validity compared with growth creative mindset.

**TABLE 1:** Descriptive statistics, psychometric properties, and latent correlations among variables (Study 1).

	Mean	*SD*	α	CR	AVE	1	2	3	4	5	6
(1) C_Fixed	3.43	1.14	0.84	0.85	0.53	/	0.24	0.41	0.00	0.00	0.01
(2) C_Growth	5.25	0.77	0.70	0.70	0.33	−0.49***	/	0.15	0.27	0.17	0.10
(3) Fixed	3.97	1.27	0.89	0.89	0.66	0.64***	−0.38***	/	0.29	0.01	0.00
(4) Growth	3.90	1.26	0.92	0.92	0.74	–0.06	0.52***	−0.54***	/	0.07	0.04
(5) CPI	4.61	0.79	0.77	0.76	0.44	0.00	0.42***	–0.08	0.26***	/	0.70
(6) CSE	4.74	0.87	0.87	0.88	0.64	–0.08	0.32***	–0.06	0.20**	0.84***	/

C_Fixed, creative fixed mindset; C_Growth, creative growth mindset; Fixed, fixed mindset of intelligence; Growth, growth mindset of intelligence; CPI, creative personal identity; CSE, creative self-efficacy; α, Cronbach’s α coefficient; CR, composite reliability; AVE, average variance extracted. Latent correlations are included below the diagonal, and shared variances are presented above the diagonal. **p < 0.01; ***p < 0.001.

Discriminant validity was evaluated by comparing CMS’s AVE with its shared variances with other relevant constructs. Specifically, discriminant validity is evident if the average variances extracted for creative mindsets exceed the shared variance between mindsets and other measures (Hair et al., 2009). As displayed in Table 1, the AVE for fixed creative mindset (AVE = 0.53) was greater than its shared variances with the two types of intelligence mindsets (*r*^2^ was 0.41 with fixed intelligence mindset or 0.00 with growth intelligence mindset), creative identity (*r*^2^ = 0.00), and CSE (*r*^2^ = 0.01). Likewise, the AVE for growth creative mindset (AVE = 0.33) was higher than all shared variances with the other four constructs (ranging from 0.10 to 0.27). The results provided empirical evidence for the discriminant validity for each creative mindset. Meanwhile, the AVE for either creative mindset was greater than their shared variance (*r*^2^ = 0.24), further supporting the two-factor model.

#### Incremental Validity

The creative mindsets were discriminated from the mindsets of intelligence, although they were correlated to a considerable extent. For example, the correlation coefficient between fixed creative mindset and fixed mindset of intelligence was 0.64, and that between growth creative mindset and growth mindset of intelligence was 0.52. It’s necessary to examine whether creative mindsets have an incremental validity beyond mindsets of intelligence in explaining creativity-related outcomes. As suggested by Hunsley and Meyer (2003), the typical approach to assess the incremental validity is by using hierarchical multiple regression analyses to determine the additional contribution of one measure in predicting the criterion after other variables have been entered into the analysis. Thus, to evaluate the incremental validity of creative mindsets in predicting a given criterion, mindsets of intelligence were entered at the first step of the regression analysis, and creative mindsets were entered at the second step. A significant R-squared change at the second step indicated a tangible incremental validity of creative mindsets since any shared variance predicting the criterion should be assigned only to creative mindsets in such an order of regression models. Stronger evidence for creative mindsets’ incremental validity was displayed when the variable entry order was flipped, i.e., creative mindsets were entered at the first step, and r-squared change due to the inclusion of intelligence mindsets at the second step was no longer significant.

We conducted hierarchical multiple regression analyses to test the incremental validity of creative mindsets in predicting CPI and CSE, two creativity-specific constructs that were associated with yet different from creative mindsets (Hass et al., 2016). At step 1, participants’ demographic variables (i.e., gender, education, and major) were entered as control variables. Mindsets of intelligence and creative mindsets were entered at the second step. At the third and final step, all variables were included in the model. The incremental validity was then inferred based on the pattern of r-squared changes at the second and third steps. As shown in Table 2, when entered in the final step, creative mindsets yielded a significant r-squared change in predicting both CPI (M3, △*R*^2^ = 0.072, Δ*F*_(2,208)_ = 9.131, *p* < 0.001) and CSE (M6, △*R*^2^ = 0.033, Δ*F*_(2,208)_ = 4.15, *p* < 0.05). However, intelligence mindsets did not yield any significant change (M3, △*R*^2^ = 0.072, Δ*F*_(2,208)_ = 0.17, *ns*; M6, Δ*R*^2^ = 0.033, Δ*F*_(2,208)_ = 0.61, *ns*) when entered in the final step. Taking all the evidence into account, it strongly indicated that creative mindsets had an incremental validity beyond mindsets of intelligence in explaining creativity-specific variables.

**TABLE 2:** Hierarchical regression results for mindsets of creativity and intelligence (Study 1).

	DV: creative personal identity				DV: creative self-efficacy			
	M1	M2a	M2b	M3^*d*^	M4	M5a	M5b	M6^*e*^
Gender^a^	0.22***	0.21**	0.18**	0.17**	0.35***	0.34***	0.33***	0.33***
Education^b^	−0.16*	–0.11	−0.13*	–0.12	–0.09	–0.05	–0.08	–0.07
Major^c^	0.07	0.06	0.06	0.06	0.02	0.01	0.01	0.00
Growth		0.19*		0.02		0.14		0.09
Fixed		0.03		–0.03		0.03		0.09
C_Growth			0.34***	0.33**			0.20**	0.18*
C_Fixed			0.12	0.13			–0.01	–0.07
*R*^2^	0.085***	0.113**	0.183***	0.185***	0.136***	0.151***	0.178***	0.183***
Δ*R*^2^		0.028*	0.098***	0.072***/0.001		0.014	0.042**	0.033*/0.005

DV, dependent variable. ^a^0 for female and 1 for male; ^b^0 for undergraduate and 1 for graduate; ^c^1 for STEM (science, technology, engineering, and math) major and 0 for the others; ^d^ΔR^2^ of M3 before or after the slash (/) was compared with M2a or M2b, respectively; ^e^ΔR^2^ of M6 before or after the slash (/) was compared with M5a or M5b, respectively. *p < 0.05; **p < 0.01; ***p < 0.001.

To summarize, employing a Chinese sample, Study 1 indicated that the CMS was of satisfactory psychometric properties in terms of reliability, construct validity, and discriminant and convergent validities. Growth creative mindset and fixed creative mindset were moderately and negatively intercorrelated but independent of each other. Creative mindsets had more power in predicting creativity-specific constructs (i.e., CPI and CSE in our study) than general mindsets of intelligence, lending further support for the discrimination between them. Moreover, consistent with prior studies (O’Connor et al., 2013; Karwowski, 2014), those who held a malleable rather than fixed mindset about creativity demonstrated higher levels of CPI and CSE. However, just like most extant research, Study 1 was based on a student sample, which limits the generalization of the findings. Thus, we decided to obtain survey data from full-time employees with the aim to generalize the findings to the real workplace.

## Study 2

In Study 2, we collected data from an independent employee sample to cross-validate the psychometric properties of the CMS and, more importantly, to examine its generalizability in the workplace. We believe it’s essential to recruit full-time employees since they are the primary creators in organizations. It is of great value to investigate how they think of the nature of creativity.

### Participants

For Study 2, we invited full-time employees from different companies in China to participate in our survey. We sent out questionnaires to 220 employees and received 205 valid responses, resulting in a response rate of 93.2%. Among the respondents, 136 (66.3%) were male, and age ranged from 21 to 62, with a mean of 31.68 years old (*SD* = 8.30). Average organizational tenure was 5.54 years (*SD* = 8.12), and average job tenure was 3.75 years (*SD* = 5.40).

### Measures

We used the same scales as those in Study 1 to measure fixed and growth creative mindsets (Karwowski, 2014), CPI (Burgmer et al., 2019), and CSE (Tierney and Farmer, 2002). Cronbach’s α was 0.88, 0.64, 0.79, and 0.87, respectively.

### Results and Discussion

As in Study 1, we relied on CFA to examine the CMS’s construct validity and to assess convergent and discriminant validity among these four constructs.

The hypothesized two-factor model in which fixed and growth creative mindsets were correlated but independent of each other had an acceptable fit with the data (χ^2^_(__34__)_ = 88.13, *p* < 0.001, CFI = 0.91, TLI = 0.88, RMSEA = 0.088, 90% CI = [0.066, 0.111], SRMR = 0.073). Conversely, the single-factor model resulted in a much worse fit (χ^2^_(35)_ = 181.24, *p* < 0.001, CFI = 0.76, TLI = 0.69, RMSEA = 0.143, 90% CI = [0.123, 0.164], SRMR = 0.113). A significant chi-square difference (Δχ^2^_(1)_ = 93.11, *p* < 0.001) lent additional support for the superiority of the two- over the one-factor model. Furthermore, a non-significant latent correlation (*r* = −0.08, *t* = −0.80, *p* = 0.426) indicated that fixed and growth creative mindsets were two independent constructs. Taking these together, the hypothesized two-factor structure was supported. Results of the two-factor model are indicated in Figure 1.

We conducted another CFA with all four constructs in the same model to assess their convergent and discriminant validity simultaneously. The hypothesized four-factor model had an excellent fit (χ^2^_(129)_ = 202.16, *p* < 0.001, CFI = 0.95, TLI = 0.94, RMSEA = 0.053, 90% CI = [0.038, 0.066], SRMR = 0.055). Latent correlations and shared variances between them, and CR and AVE for each construct, are shown in Table 3. All but growth mindset had CR higher than 0.70 and AVE close to or greater than 0.50, indicating acceptable convergent validity according to the criterion suggested by Hair et al. (2009). Moreover, AVE of each construct was higher than its shared variances with the remaining three constructs, providing empirical evidence for adequate discriminant validity.

**TABLE 3:** Descriptive statistics, psychometric properties, and latent correlations among variables (Study 2).

	Mean	*SD*	α	CR	AVE	1	2	3	4
(1) C_Fixed	3.27	1.35	0.87	0.87	0.57	/	0.01	0.01	0.03
(2) C_Growth	5.20	0.89	0.64	0.64	0.27	−0.02	/	0.03	0.05
(3) CPI	3.36	0.70	0.79	0.79	0.49	−0.14*	0.13	/	0.67
(4) CSE	3.43	0.74	0.87	0.87	0.63	−0.14*	0.18*	0.61**	/

Latent correlations are included below the diagonal, and shared variances are presented above the diagonal. *p < 0.05; **p < 0.01.

It’s worth noting that, similar to results of Study 1, the psychometric properties of the growth creative mindset were not as good as those of the fixed creative mindset. It was due to relatively smaller factor loadings of growth mindset items compared with those of fixed mindset items, though all loadings were significant. Thus, we decided to include another employee sample to further investigate the CMS’s psychometric properties. More importantly, we extended our scope to test whether creative mindsets were of predictive validity. Specifically, we aimed to explore whether measures of fixed and growth creative mindsets were associated with an employee’s creative performance in the workplace.

## Study 3

Study 3 aimed to explore how this belief about creativity in turn influences creative performance, and to reveal its underlying mechanism. Preliminary research suggests that creative mindsets are associated with an individual’s creative performance. For example, Karwowski (2014) found that the fixed creative mindset was associated with lower performance in insight-problem solving. In contrast, although the relationship between a growth creative mindset and creative performance was positive, it was not significant. The findings were replicated in Intasao and Hao’s (2018) recent study, which recruited high school students to complete a divergent thinking task. Specifically, only a fixed mindset was significantly (and negatively) related to flexibility and originality, the two most typical measures of creativity for divergent thinking tasks. Based on a college student sample, Royston and Reiter-Palmon (2017) also found that a fixed creative mindset was significantly associated with lower performance in a problem-solving task. Differently from findings of the two aforementioned studies, however, a malleable creative mindset was positively related to creative performance in their study. O’Connor et al. (2013) provided convergent evidence for the detrimental effect of a fixed creative mindset on creative performance across three studies. They demonstrated that college students who held a trait-like fixed creative mindset, or those who were primed to temporarily endorse the fixed mindset, exhibited poorer performance in the subsequent divergent thinking task. Participants in this study who held a fixed mindset also self-reported a lower level of overall or domain creativity.

However, the existing studies suffered from several limitations. First, more often than not, high school or college students were recruited as the participants. Second, participants were required to solve creative tasks that were of no relevance to the actual jobs they were supposed to undertake in real life or at work. Third, in studies (e.g., O’Connor et al., 2013) where the situation was relevant to real life, creative performance was measured by participants’ self-report. To echo Hass et al. (2017), we believe it’s necessary to explore to what extent creative mindsets are related to actual creative performance in real work settings, and to remedy the bias of self-report of creativity.

Building on extant research of implicit theory (Dweck, 1986; Dweck et al., 1995, 2003), and implicit theory of creativity in particular (O’Connor et al., 2013; Karwowski, 2014; Royston and Reiter-Palmon, 2017), we postulate that a growth creative mindset boosts, while a fixed creative mindset hinders, creativity through the mediating mechanism of the effort individuals put into their work. The critical difference between individuals holding a growth versus a fixed mindset lies in their belief in the role of effort in achieving desired goals. Individuals holding a growth mindset believe that one can always overcome setbacks and achieve goals by exerting sufficient effort into their work (Dweck, 2006). On the contrary, people who adopt the fixed mindset believe that one’s achievement is a function of his/her innate ability and thus tend to deny the merit of hard work (Dweck, 1986; Dinger and Dickhäuser, 2013). As a result, individuals holding a growth mindset outperform their counterparts holding a fixed mindset in terms of achievements, especially when both are under adverse situations or facing setbacks (Claro et al., 2016; Sisk et al., 2018). It’s not surprising to see that most mindset intervention programs start with changing participants’ negative effort beliefs (Paunesku et al., 2015; Yeager et al., 2016, 2019; Dweck and Yeager, 2019).

Besides, we further explore the underlying mechanism of creativity, drawing upon the creative behavior as agentic action (CBAA) model (Karwowski and Beghetto, 2019). The core premise of the CBAA model is that people’s creative self-beliefs help to determine their engagement in transferring creative potential to creative behavior, and this process is called agentic action. To what extent one person is willing to engage in creative activities is a function of creative self-confidence and of the perceived value of creativity (Karwowski and Beghetto, 2019). Accordingly, we speculate that how much effort, or the amount of time, hard work, and perseverance (VandeWalle et al., 1999), one will devote to creative activities will depend on his/her belief of creativity. Hard work, perseverance, and persistency are also necessities for creativity. Theory and empirical research have convincingly indicated that effort is one of the key determinants of creative performance (De Dreu et al., 2008; Nijstad et al., 2010; Baas et al., 2013). It’s mainly because at the early stage of ideation, creators usually come up with common, ordinary ideas. Highly novel and useful ideas or solutions can be achieved only after we have exhausted the easily accessible idea pool and continue to engage in in-depth exploration. Creators with a growth mindset are more likely to be persistent in the whole creative process, as they recognize the importance of continuous effort for creative outputs. Recently, several studies have shown that individuals with a growth creative mindset do exert more effort, experience more positive affect and less negative affect, and have more interest when engaging in creative activities (O’Connor et al., 2013; Puente-Díaz and Cavazos-Arroyo, 2017a; Intasao and Hao, 2018). However, in the study of Intasao and Hao (2018), effort did not serve as a mediator between creative mindsets and creative performance, because effort was not significantly related to the latter. One possible explanation might be that they only recruited student participants to complete *ad hoc* tasks, which were of little relevance to their actual job duty. The function of effort may not be fully revealed in such a time-limited and context-free situation.

In Study 3, we relied on a full-time employee sample to test the mediating role of effort. Since employees with a growth creative mindset have faith in the effect of prolonged and motivated efforts, they are willing to endeavor throughout the process of trial and error. As a result, they are more likely to obtain superior creative performance. On the contrary, those with a fixed creative mindset believe that creativity is innate and can’t be changed no matter how hard they try. Not surprisingly, they exert less effort and thus lower the possibility of achieving better creative performance. Therefore, we came up with two hypotheses, as below:

Hypothesis 1. Effort will mediate the positive effect of a growth creative mindset on creative performance.

Hypothesis 2. Effort will mediate the negative effect of a fixed creative mindset on creative performance.

### Participants

For Study 3, our sample consisted of real estate agents in China. Like many other sales representatives, real estate agents rely heavily on their creativity to survive and thrive in a tough and highly competitive workplace (Gong et al., 2009; Hirst et al., 2015). In order to achieve assigned goals efficiently, they need to display creativity by identifying appropriate ways to connect and interact with clients, developing new ways to peddle properties, and adopting strategies to facilitate client sales. They provide us a good sample to investigate the relationship between mindsets and creativity. We sent out questionnaires to 304 real estate agents and received a valid response from 282 employees, resulting in a response rate of 92.8%. Among the respondents, 133 (46.8%) were male, and age ranged from 20 to 41, with a mean of 27.36 years old (*SD* = 3.57). Average organizational tenure was 1.26 years (*SD* = 1.41), and average job tenure as a salesperson was 3.76 years (*SD* = 3.01). To eliminate common method bias, we invited the agents’ immediate supervisors to rate each agent’s creativity.

### Measures^1^

#### Creative Mindsets

We used the same scale as in Studies 1 and 2, Karwowski’s (2014) 10-item CMS. For Study 3, Cronbach’s α was 0.87 for fixed creative mindset and 0.79 for growth creative mindset.

#### Effort

Following the previous research (VandeWalle et al., 1999), we measured effort by six items that asked employees to report how much time, work intensity, and behavioral persistence they had put into work. Example items are “I put in long hours throughout the project” (time), “I strive as hard as I can to be successful in this project” (work intensity), and “I do not give up at all throughout the whole project” (behavioral persistence). Participants responded on a seven-point Likert scale (1 = strongly disagree, 7 = strongly agree; α = 0.83).

#### Creativity

Supervisors rated creativity on a four-item scale by Gong et al. (2009), which was developed in order to measure sales agents’ creativity based on focus group interviews with them. A sample item is “This person often uses creativity to develop new clients through different means and channels.” Supervisors rated agents on a seven-point Likert scale (1 = never, 7 = almost; α = 0.89).

### Results and Discussion

#### Confirmative Factor Analysis

Again, we conducted a series of CFAs to examine the construct validity of the CMS as in Studies 1 and 2. The two-factor model fit the data well (χ^2^_(34)_ = 72.34, *p* < 0.01, CFI = 0.96, TLI = 0.95, RMSEA = 0.063, 90% CI = [0.043, 0.083], SRMR = 0.049). As shown in Figure 1, factor loadings ranged between 0.60 and 0.81 and were all significant at the 0.001 level, suggesting good construct validity. Growth and fixed mindsets were slightly and negatively correlated (*r* = −0.21, *t* = −3.05, *p* < 0.01), which indicated that they were distinct from each other. The alternative one-factor model fell far below the acceptable goodness-of-fit criterion (χ^2^_(35)_ = 405.38, *p* < 0.001, CFI = 0.64, TLI = 0.53, RMSEA = 0.194, 90% CI = [0.177, 0.211], SRMR = 0.163). A significant chi-square change (Δχ^2^_(1)_ = 333.04, *p* < 0.001) between these two models further supported the hypothesized two-factor structure.

#### Mechanism of the Effects of Creative Mindsets on Creativity

**FIGURE 2:**
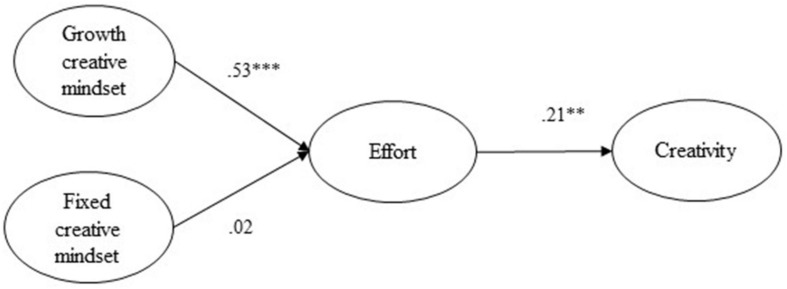
Structural equation modeling (SEM) results for creative mindsets, effort, and creativity (Study 3). ^∗∗^*p* < 0.01; ^∗∗∗^*p* < 0.001. Circles are used to indicate that all four variables were constructed as latent in SEM. For clarity, indicators of each latent variable are intentionally omitted from the figure.

We conducted structural equation modeling (SEM) to examine how the two types of creative mindsets influenced creativity through the mediating effect of effort. The mediation model fit the data well (χ^2^_(164)_ = 323.29, *p* < 0.001, CFI = 0.94, TLI = 0.93, RMSEA = 0.059, 90% CI = [0.049, 0.068], SRMR = 0.048). Creative growth mindset was significantly and positively related to effort (γ = 0.53, *t* = 9.51, *p* < 0.001), but fixed mindset was not (γ = 0.02, *t* = 0.27, *p* = 0.79). Effort was significantly and positively associated with supervisor-rated creativity (β = 0.21, *t* = 3.38, *p* < 0.01). Based on the path coefficients, we calculated and further tested the indirect effects using a bootstrap method with 10,000 replications (Hayes, 2013). Bootstrap results showed that the indirect effect of growth mindset on creativity was significant (*b* = 0.124, *SE* = 0.058, 95% CI = [0.011, 0.238]), but that of fixed mindset was not (*b* = 0.004, *SE* = 0.019, 95% CI = [−0.033, 0.041]). Thus, hypothesis 1 was supported, while hypothesis 2 was not. SEM results are presented in Figure 2.

Using a full-time employee sample, the two-factor structure of the CMS was again confirmed according to CFA results. All its factor loadings were significant and above 0.60, suggesting an even better construct validity than in Studies 1 and 2. Again, growth and fixed creative mindsets were distinct from each other, exhibiting moderately negative correlations. Their indirect predictive effects on creativity were also distinct but not opposite as expected—people with malleable perceptions of creativity put in more effort, which thus led to a higher level of creative performance in the workplace, while those with a fixed belief didn’t show significant variance in their efforts or creativity. This might indicate that the latter would not necessarily reduce effort, though they are by no means willing to make an extra effort. In their opinion, there is no chance for someone to achieve optimal creative performance through hard work and persistence. Overall, we verified the validity and reliability of the CMS with another vocational sample and further explored its predictive effects on employee creativity in a real workplace. A path analysis revealed the mediating role of effort, underlying the mechanism between growth creative mindsets and creativity.

## General Discussion

Using three independent samples, we cross-validated the psychometric properties of the CMS and tested its applicability in the Chinese context. Results indicated that a two-factor structure, where growth mindset and fixed mindset emerged as two correlated yet independent dimensions, fit the data well for both college student and full-time employee samples. Measures of both types of creative mindsets met the psychometric requirements in terms of reliability (internal consistency) and validity (construct, convergent, and discriminant validities). Moreover, our study provided evidence for the incremental validity of creative mindsets over mindsets of intelligence in explaining CPI and CSE. Finally, our study demonstrated that measures of creative mindsets could predict employees’ creative performance as rated by their supervisors, indicating their generalizability to the real workplace.

It’s worth noting that the psychometric properties of the growth creative mindset were not as good as those of the fixed creative mindset, especially for Studies 1 and 2. Factor loadings of growth mindset items were relatively smaller than those of fixed mindset items, though all loadings were significant. Actually, quite a few studies adopting the CMS had a similar finding that certain items were somewhat weaker indicators of a growth mindset (Karwowski, 2014; Hass et al., 2016; Tang et al., 2016; Karwowski et al., 2019b). Karwowski (2014) and Karwowski et al. (2019a) attributed this to the possibility that growth mindset items were more susceptible to social desirability bias. Researchers have adopted two approaches to avoid this potential measurement bias. Based on the unidimensional assumption, the first approach was to only include fixed mindset items (e.g., O’Connor et al., 2013). In doing so, a measure of fixed or growth mindset was operationalized as a low or high score on the single measurement scale. Other researchers employed a *post hoc* approach to delete the item if its factor loading on the assumed construct was below a certain value (e.g., Hass et al., 2016; Intasao and Hao, 2018). However, the threshold of factor loading that triggered item deletion was arbitrary and inconsistent because it was usually judged according to rules of thumb. For example, Hass et al. (2016) deleted one item whose loading was smaller than 0.40, and Intasao and Hao (2018) dropped one item with a loading below 0.35.

In our study, we decided to measure both types of creative mindsets and kept all 10 items of the CMS for two reasons. Firstly, despite one item having relatively small loading, the measure of growth mindset had acceptable reliability (Cronbach’s α = 0.70, CR = 0.70). Moreover, low factor loading disappeared in Study 3 (see Figure 1), indicating that the error pertaining to growth mindset measurement might be random rather than systematic. Secondly, the hypothesized relationships between creative mindsets and the outcome variables were consistently supported across the three studies. Specifically, a growth creative mindset was significantly and positively associated with CPI, CSE, and supervisor-rated creativity, while a fixed mindset was not. Taken together, these suggested that occasionally low factor loading of CMS items was not a major concern in exploring the effects of creative mindsets on individuals’ creative behavior and performance.

### Theoretical Contributions and Practical Implications

The current study has several contributions to the creative mindset literature. First and foremost, we cross-validate the psychometric properties of the newly constructed CMS in the Chinese setting. We echo other researchers that the CMS is applicable in both Western (e.g., Karwowski, 2014; Hass et al., 2016; Tang et al., 2016; Royston and Reiter-Palmon, 2017) and Eastern (e.g., Lee and Choi, 2014; Intasao and Hao, 2018) cultures. Researchers can rely on this scale to further explore the nature, antecedents, and consequences of implicit theories of creativity within a specific cultural context or cross-culturally.

Second, and more importantly, our study is among the first to examine the generalizability of creative mindsets’ effect on creativity to the real work place. As mentioned above, extant studies adopt the decontextualized approach by revealing the effects of creative mindset on participants’ (mainly students) creative performance on *ad hoc* tasks (e.g., unusual uses task) that are irrelevant to their actual job duty. Our study implies that creative mindset also matters for full-time employees in the real workplace, since those holding a growth creative mindset are more likely to receive a better evaluation of creativity from their immediate supervisors. Our study pioneers in this context-embedded investigation, and more research is needed.

Third, we empirically differentiate creative mindsets from mindsets of intelligence by comparing their incremental effects in predicting creativity-specific constructs. While research has indicated that implicit theory of creativity is conceptually different from that of intelligence for most people (e.g., Sternberg, 1985; Plucker et al., 2017), our study provides direct empirical evidence that creative mindsets have unique and incremental power in predicting creativity-specific constructs (i.e., CPI and CSE in the current study) beyond intelligence mindsets. By doing so, we lend support to the psychometric soundness of the CMS in capturing the unique nature of creative mindsets.

Fourth and last, the current study advances our understanding of the underlying mechanism linking creative mindsets to creativity. Although it has long been pointed out by Dweck (1986, 2006) that the attitude toward effort differentiates people with a growth mindset from those holding a fixed mindset, the mediating role of effort between creative mindsets and creative performance is yet to be confirmed. Our study initially addresses this issue. It reveals that effort is the behavioral mechanism through which individuals who hold a growth creative mindset can achieve desirable creative outcomes.

### Practical Implications

Our study also provides implications for future practice both in academic research and work settings. First of all, based on three independent samples, the validation of the CMS is therefore conclusive. Researchers and practitioners can rely on this scale to further investigate people’s creative beliefs, thus answering intriguing questions like the relationship between creative beliefs and creativity.

Moreover, the result of our study prompts that the CMS should be used with caution. There is a sign of low factor loading of certain CMS items, reminding our fellow researchers to attend to a possible susceptibility of growth mindsets to social desirability bias (Karwowski, 2014; Karwowski et al., 2019a). Taking actions like anonymity may help to reduce the bias.

Finally, our findings offer insights for the design of creativity enhancement programs. According to the results of our study, the key to obtain high creative performance lies in the malleable belief that sustaining effort brings in creativity. Thus, fostering an appropriate attitude toward effort should be an essential element of such programs. Specifically, in R&D units or educational institutions where people value and cultivate creativity, the growth creative mindset can be a starting point to lever creative outcomes via sustained effort.

### Limitations and Future Directions

Despite providing consistent evidence for the psychometric features of the CMS across two studies using different samples, the present study has a few limitations. The first limitation is due to the cross-sectional design. In order to provide additional support for Studies 1 and 2, which relied entirely on self-report data, in Study 3, we collected multisource data from both employees and their supervisors to minimize the common method bias. As it was limited by the cross-sectional nature, however, it is not possible to infer a causal relationship between creative mindsets and creative performance. Longitudinal or experimental research is needed to determine the causal effects of creative mindsets on creative outcomes.

Another limitation stems from the neglect of boundary conditions of the relationship between creative mindsets and creative performance. As the research of creative self-beliefs evolves, the role of creative mindsets in the bigger picture is emerging. It’s important to delineate how creative mindsets and other self-belief constructs (e.g., CPI and self-efficacy in this study) intersect in explaining creativity (Karwowski and Beghetto, 2019). We believe that it would be of value, both theoretically and practically, to study the creative mindsets under a more integrative perspective. For example, to complete theoretical models like the CBAA (Karwowski and Beghetto, 2019) with creative mindsets may enhance our understanding of the role mindsets play in linking creative potentials to outcomes. Besides, according to the implicit theory of mindsets (Dweck, 2006), negative experiences like being in adverse situations or facing setbacks enhance the effects of mindsets, such that people with a growth mindset are more likely to achieve desirable creative outcomes at work than those bearing fixed mindsets (Claro et al., 2016; Sisk et al., 2018). Evidence from educational studies provides some insights (for meta-analysis, see Sisk et al., 2018), yet less attention has been paid to creativity study. More work needs to be done to have a better knowledge of how creative mindsets influence creativity in different contexts.

Last but not least, future study should explicate why the reliability and validity of the growth creative mindset appear to be lower than those of the fixed creative mindset. As suggested in previous studies (e.g., Dweck et al., 1995; Karwowski et al., 2019a), growth mindset items were presumed to be more prone to social desirability. However, we didn’t empirically test this assumption in our study. We believe in the necessity of solving the puzzle and encourage researchers to explore the effect of social desirability, among other possible factors, on measurements of creative mindsets.

## Data Availability Statement

The data sets generated for this study are available on request to the corresponding author.

## Ethics Statement

This study was carried out in accordance with the recommendations of the research ethics guidelines of the Institutional Review Board of the Institute of Psychology, Chinese Academy of Sciences, with written informed consent from all subjects. All subjects gave written informed consent in accordance with the Declaration of Helsinki. The protocol was approved by the Institutional Review Board of the Institute of Psychology, Chinese Academy of Sciences (Protocol Number: H17002).

## Author Contributions

XB designed this study. YZ and XB collected and analyzed the data. YZ, WY, and XB wrote the manuscript.

## Conflict of Interest

The authors declare that the research was conducted in the absence of any commercial or financial relationships that could be construed as a potential conflict of interest.

## Footnotes

1 All scales used in Study 1–3 are available as online Supplementary Materials.
