# Supplementary material for: Creative Mindsets: Scale Validation in the Chinese Setting and Generalization to the Real Workplace
Source: Front Psychol. 2020 Mar 26;11:463. doi: 10.3389/fpsyg.2020.00463 (PMC7113404; doi:10.3389/fpsyg.2020.00463)
Supplement: Supplementary file 1 [file Data_Sheet_1.docx]

Supplementary Material

# Scales used in the study

## **Creative mindsets scale** (Study 1-3, Karwowski, 2014)

| *Growth creative mindsets* |
| --- |
| 1. Everyone can create something great at some point if he or she is given appropriate conditions |
| 3) Anyone can develop his or her creative abilities up to a certain level |
| 5) Practice makes perfect—perseverance and trying hard are the best ways to develop and expand one’s capabilities |
| 7) Rome wasn’t built in a day—each creativity requires effort and work, and these two are more important than talent |
| 9) It doesn’t matter what creativity level one reveals—you can always increase it |
| *Fixed creative mindsets* |
| 2) You either are creative or you are not—even trying very hard you cannot change much |
| 4) You have to be born a creator—without innate talent you can only be a scribbler |
| 6) Creativity can be developed, but one either is or is not a truly creative person |
| 8) Some people are creative, others aren’t—and no practice can change it |
| 10) A truly creative talent is innate and constant throughout one’s entire life |

1= strongly disagree, 7 = strongly agree.

## **Mindset about intelligence** (Study 1, Dweck, 2000)

| 1. You have a certain amount of intelligence, and you can’t really do much to change it. |
| --- |
| 1. Your intelligence is something about you that you can’t change very much. |
| 1. No matter who you are, you can significantly change your intelligence level. |
| 1. To be honest, you can’t really change how intelligent you are. |
| 1. You can always substantially change how intelligent you are. |
| 1. You can learn new things, but you can’t really change your basic intelligence |
| 1. No matter how much intelligence you have, you can always change it quite a bit. |
| 1. You can change even your basic intelligence level considerably. |

1= strongly disagree, 7 = strongly agree.

## **Creative identity** (Study 1 & 2, Burgmer, Forstmann, & Stavrova, 2019)

| 1. I often think about being creative. |
| --- |
| 1. My friends think of me as a creative person. |
| 1. My friends think that creativity is important to me. |
| 1. To be a creative person is an important part of my identity. |

1= strongly disagree, 7 = strongly agree.

## **Creative efficacy** (Study 1 & 2, Tierney & Farmer, 2002)

| 1. I have confidence in my ability to solve problems creatively |
| --- |
| 1. I feel that I am good at generating novel ideas |
| 1. I am good at coming up with new ideas of my own based on others |
| 1. I am good at generating novel solutions to solve problems |

1= strongly disagree, 7= strongly agree

## **Effort** (Study 3, VandeWalle, Brown, Cron, & Slocum, 1999)

| 1. I put in long hours throughout the project. |
| --- |
| 1. My clients know me by the long hours I keep. |
| 1. Throughout the whole project, I really exert myself to the fullest. |
| 1. I strive as hard as I can to be successful in this project. |
| 1. I do not give up at all throughout the whole project. |
| 1. I work untiringly at interacting with the client until I get the project done. |

1= strongly disagree, 7= strongly agree

## **Creativity performance** (Study 3, Gong, Huang, & Farh, 2009)

| 1. This person often develops creative custom-made product/service packages for clients. |
| --- |
| 1. This person often uses creativity to develop new clients through different means and channels. |
| 1. This person often uses creativity to increase sales forces in different ways. |
| 1. This person often develops creative methods for promotion and sales. |

1= never, 7 = almost
